# Development of plasma cell myeloma in a B-cell chronic lymphocytic leukemia patient with chromosome 12 trisomy

**DOI:** 10.1186/1756-0500-6-433

**Published:** 2013-10-29

**Authors:** Welbert de Oliveira Pereira, Nydia Strachman Bacal, Rodolfo Patussi Correia, Ruth Hissae Kanayama, Elvira Deolinda Veloso, Daniela Borri, Nelson Hamerschlak, Paulo Vidal Campregher

**Affiliations:** 1 Instituto Israelita de Responsabilidade Social, Instituto Israelita de Ensino e Pesquisa, Hospital Israelita Albert Einstein, São Paulo, Brasil; 2 Laboratório de Citometria de Fluxo, Laboratório Clínico, Hospital Israelita Albert Einstein, 627, Albert Einstein Av, São Paulo, Brasil; 3 Laboratório de Genética, Laboratório Clínico, Hospital Israelita Albert Einstein, São Paulo, Brasil; 4 Departamento de Hematologia e Hemoterapia, Hospital Israelita Albert Einstein, São Paulo, Brasil; 5 Centro de Hematologia de São Paulo, São Paulo, Brazil; 6 Laboratório de Biologia Molecular, Laboratório Clínico, Hospital Israelita Albert Einstein, São Paulo, Brasil

**Keywords:** B-Cell chronic lymphocytic leukemia, Plasma cell myeloma, Chromosome 12 trisomy

## Abstract

**Background:**

Cancer development results from the progressive accumulation of genomic abnormalities that culminate in the neoplastic phenotype. Cytogenetic alterations, mutations and rearrangements may be considered as molecular legacy which trace the clonal history of the disease. Concomitant tumors are reported and they may derive from a common or divergent founder clone. B-cell chronic lymphocytic leukemia (B-CLL) and plasma cell myeloma (PCM) are both mature B-cell neoplasms, and their concomitancy, albeit rare, is documented.

**Case presentation:**

Here, we described a patient with prior B-CLL with secondary development of PCM. Cytogenetic and multi parametric flow cytometry analyses were performed. The B-CLL population presented chromosome 12 trisomy, unlikely the arisen PCM population.

**Conclusion:**

The close follow up of B-CLL patients is important for early intervention in case of development of other malignancy, such as myeloma. Our observation suggests these two diseases may have arisen from different clones. We understand that the investigation of clonal origin may provide important information regarding therapeutic decisions, and should be considered in concomitant neoplasm.

## Background

It has been demonstrated that cancers are composed of heterogeneous malignant cell populations, harboring distinct sets of genomic aberrations such as mutations, copy number alterations, chromosomes abnormalities etc. [1-4]. Another phenomenon described in hematological malignancies is lineage plasticity [5], in which two different malignancies originate from the same malignant cell.

B-cell chronic lymphocytic leukemia (B-CLL) and plasma cell myeloma (PCM) are mature B-cell neoplasms, and their concomitancy in a patient, although rare, has been previously reported [6-10]. Whether the same B-cell originates both malignancies, in cases of concomitancy, is a matter of controversy.

Here, we report a patient with a diagnosis of B-CLL that developed concomitant PCM 6 years after leukemia diagnosis. We characterized the two malignant populations by multi parametric flow cytometry and performed cytogenetic analysis.

## Case presentation

A 60 year-old female patient was diagnosed with B-CLL in 2005 with trisomy of the chromosome 12, revealed by cytogenetic analysis. Plasma cell abnormalities were not reported. The patient was treated with intermittent cycles of chlorambucil and prednisone. On March 2011 the patient developed anemia and thrombocytopenia and on April 2011 she was seen at our institution for a diagnostic workup.

There was no evidence of lymphadenopathy or hepathosplenomegaly at the physical evaluation. Complete blood counts revealed: hemoglobin: 8.6 g/dL; leucocytes: 1,300 cells/uL; neutrophils: 46.4%; lymphocytes: 38.6%; monocytes: 13.2%; platelet count: 17,000/uL. Bone marrow aspiration, biopsy, cytogenetic analysis and immunophenotyping were performed. It was observed 37.2% of plasma cells and 6.8% of lymphocytes according to the myelogram. Bone marrow cytogenetic analysis revealed 47, XX, +12 [9] /46,XX [11]. Bone marrow immunophenotyping revealed 25% of clonal plasma cells (CD38+; CD56+; CD117+, IgG kappa+; CD19-; CD20-, CD45-; lambda-) and 13% of abnormal B Lymphocytes (CD5+/CD23+/CD19+/dim CD20+/CD38+/HLA-DR+/surface IgM and IgD kappa+). Serum immunofixation revealed IgG concentration of 3,990 mg/dL (normal range – 700 to 1,600 mg/dL). Serum protein electrophoresis uncovered an IgG monoclonal peak. The patient was diagnosed with concomitant CLL and IgG PCM. The patient was treated with four cycles of rituximab, cyclophosphamide, bortezomib and dexamethasone with partial improvement of the cytopenias.

**Figure 1:**
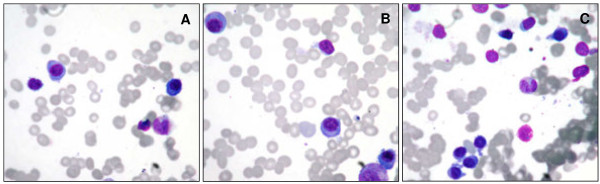
**Identification of B-CLL and abnormal plasma cell population by morphology analysis.** Bone marrow smears were dyed with Rosenfeld’s staining and morphology analysis was performed in optical microscope. The panels **A**, **B** and **C** show the morphological aspects of the B-CLL and plasma cell myeloma populations.

Another bone marrow study was done in June 2012. Myelogram revealed 33.2% of lymphocytes and 18% of plasma cells. Morphological analysis revealed small lymphocytes presenting clumped chromatin and scant cytoplasm, suggestive aspect of B-CLL. In addition, to the high frequency of the plasma cells in the bone marrow, some of them presented binucleated morphology, high nucleus/cytoplasm ration, basophilic cytoplasma and eccentric nucleus (Figure 1). Immunohistochemical analysis of bone marrow trephine biopsy showed infiltration with plasma cells (20%) expressing CD138, kappa and cyclin D1, and 10% of B-lymphocytes expressing CD5 and CD23. At that point, we decided to investigate the clonal origin of B-CLL and PCM.

**Figure 2:**
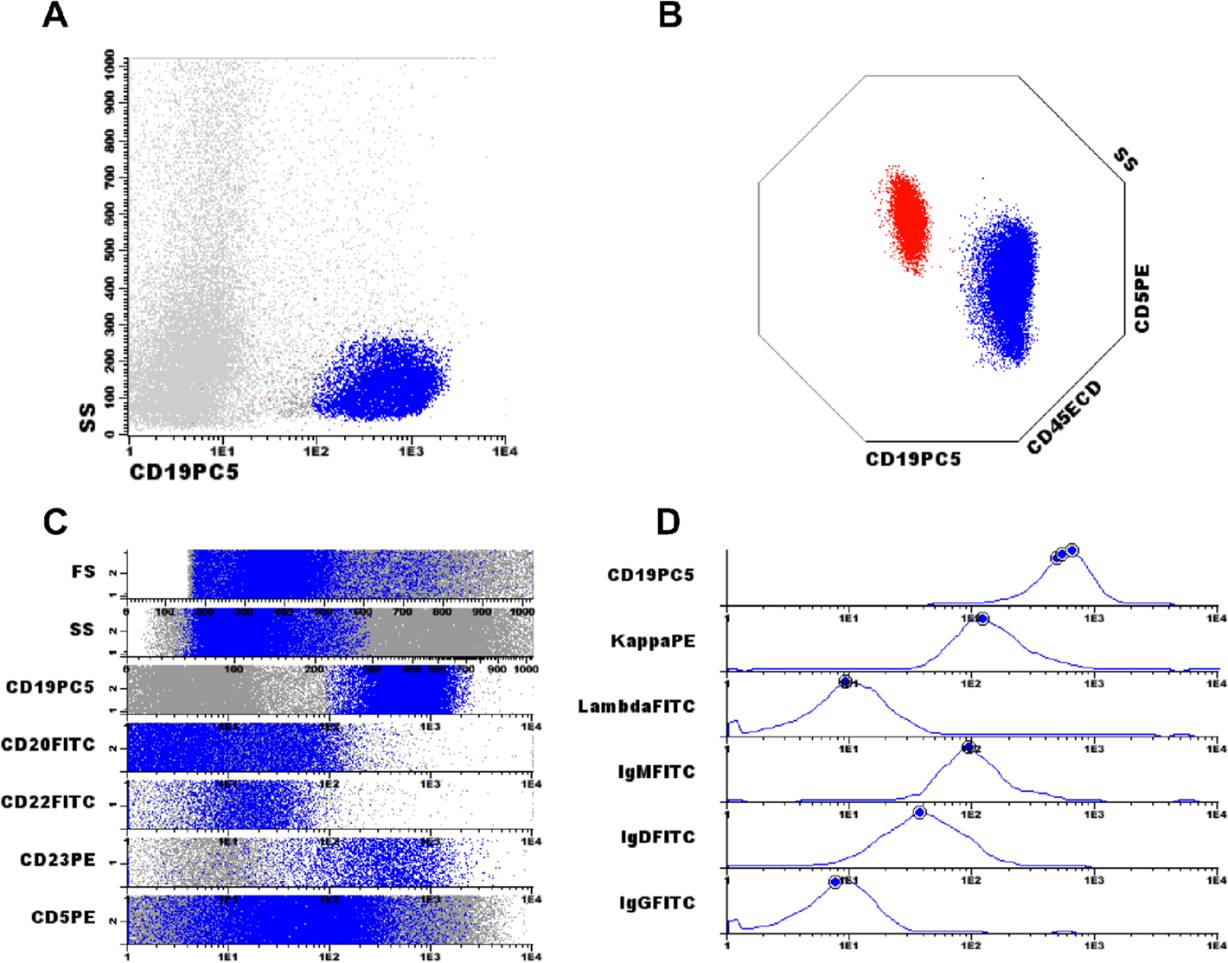
**Characterization of the B-CLL population by flow cytometry immunophenotyping. A**: The B cell population was gated by CD19 expression. **B**: Multiparametric dot plot showing T cell population (red), which is positive to CD5, and gated CD19+ B cells (blue), which presented low but positive aberrant CD5. **C**: Multiparametric dot plot in bands showing the expression of a panel of markers in gated B cells (blue dots). **D**: Multiparametric histograms confirming the monoclonality of the gated B cell population. Circles represent medians. Infinicyt Flow Cytometry software (Cyotgnos, Salamanca, Spain) was used in the analysis.

**Figure 3:**
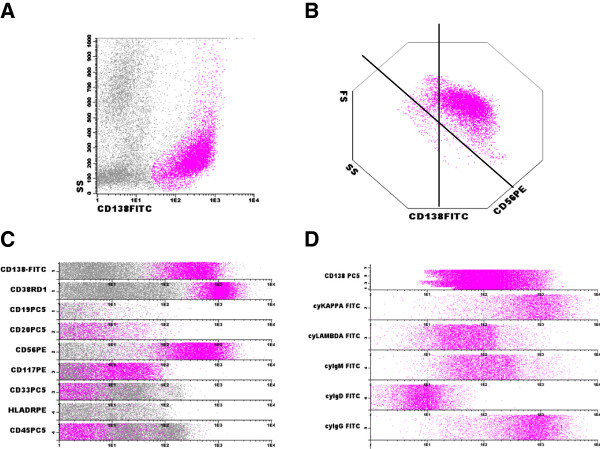
**Characterization of the discrasic plasma cell population by flow cytometry immunophenotyping. A**: The plasma cell population was gated by CD138 expression. **B**: Multiparametric dot plot showing the aberrant expression of CD56 in all gated plasma cells. **C**: Multiparametric dot plot in bands showing the expression of a panel of markers in gated plama cells (pink dots). **D**: Multiparametric dot plot in bands confirming the monoclonality of the gated plasma cell population. Infinicyt Flow Cytometry software (Salamanca, Spain) was used in the analysis.

Using multi parametric flow cytometry, we found that CD19(+) B-cells expressed CD5 (dim), CD20 (dim), CD23, CD38, kappa light chain restriction, surface IgM and IgD, and also were negative for CD10, CD22, FMC7 and lambda, compatible with a diagnosis of B-CLL (Figure 2). Gating CD138 positive cells, we identified the following pattern of expression on plasma cells: CD38(+), CD56(+), cytoplasmatic kappa light chain(+), cytoplasmatic IgG(+), and CD19(−), CD20(−), CD117(−),CD33(−), HLA-DR(−), and CD45(−) (Figure 3). The diagnosis of PCM was confirmed with clinical evaluation and complementary exams (not shown). These data confirmed the presence of concomitant B-CLL and PCM in this patient.

**Figure 4:**
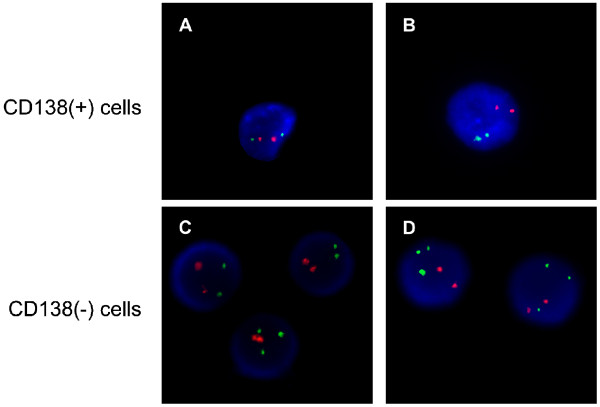
**Identification of chromosome 12 trisomy in B-CLL cells, but not in myeloma cells by FISH.** Dual color FISH for the genes ETV6 and RUNX1, whose probes detect the chromosome 12 (green) and chromosome 21 (red) respectively. **A** and **B**: Myeloma plasma cells presented two copies of each chromosome, thus negative for the trisomy. **C** and **D**: Nucleus carrying chromosome 12 trisomy were detected in 91% the cells in the CD138(−) fraction.

Next, we investigated if a prior B-CLL clone could have derived the PCM secondary disease. Since the patient had a history of previous B-CLL with chromosome 12 trisomy, and this abnormality had already been reported, albeit rarely, in PCM patients [11], we decided to study whether the same cytogenetic abnormality was also present in the PCM fraction. In order to isolate the plasma cells, we performed a bone marrow magnetic bead selection protocol using Human Whole Blood CD138 Selection Kit (Easysep, Stem Cell Technologies), obtaining CD138(+) and CD138(−) fractions. After that, we analyzed the presence of chromosome 12 trisomy in both samples by fluorescent in situ hybridization (FISH) using the kit LSI ETV6(TEL)/RUNX1(AML1) ES DC (Vysis, Inc). The analysis showed that 91% of the CD138(−) cells were positive for chromosome 12 trisomy, confirming the presence of this cytogenetic aberration in B-CLL cells. However, all cells from the CD138(+) fraction presented no additional copy of the chromosome 12 (Figure 4). Taken together, these observations suggest that the PCM cells may not have derived from a B-CLL clone, since the plasma cells did not carry the same cytogenetic abnormality present in the CLL cells.

## Conclusions

Concomitant B-CLL and PCM is rare, and the few published cases are controversial regarding the clonal origin of the two diseases. Considering that plasma cells may develop from follicular B cells, marginal-zone B cells, B-1 cells and also from memory B cells [12], it is possible that malignant plasma cells may have derived from the B-CLL clone. Thus, in theory, under some still unknown stimuli, a B cell from a patient with CLL could differentiate into a plasma cell, generating a new malignant disease.

The first publication of these concurrent neoplasias described a B-CLL patient who developed a plamacytoma. The authors suggested a common clonal origin based only on the presence of the same heavy and light chains in the two diseases [8]. One year later, Fermand and colleagues investigated one case of B-CLL and PCM producing IgG1 kappa and IgA kappa, respectively [7]. Using a more elegant approach, they cultured in vitro the B-CLL cells and induced differentiation to plasma cells, which in turn produced IgG and IgA. These data allowed them to suggest that, under some circumstances, PCM may be originated from B-CLL cells and conserve the produced immunoglobulins. The theory of one clone for these two concomitant diseases was strongly supported by immunoglobulin gene rearrangement investigation. Saltman and colleagues found the same rearrangements in B cells from blood and bone marrow plasma cells, despite different immunoglobulins isotypes [13]. One criticism to this work is the possible cross contamination of the bone marrow plasma cells with B cells from the blood, since they performed no cell sorting approach.

On the other hand, distinct clonal origins for these concomitant hematological neoplasias have been equally proposed since 1985, based on the expressed immunoglobulin chains [9] and later, based on the analysis of B cell receptor rearrangement [14]. Patriarca and coworkers reported a case of separate clonal evolution of PCM (kappa light chain) after fludarabine treatment for the B-CLL (lambda light chain), besides the two different rearrangements of heavy chain immunoglobulin detected in bone marrow, providing evidences of two clones [10].

Here, we described a case of B-CLL with chromosome 12 trisomy that developed concomitant PCM. We performed a strict flow cytometry analysis to characterize the two diseases, which revealed a B-CLL population expressing IgM and IgD kappa on the surface of B cells, and the PCM population expressing cytoplasmic IgG kappa. Differential diagnosis, such as CLL with plasmacytic differentiation and CD5+ lymphoplasmacytic lymphoma, were excluded due to: i) the clear presence of two distinct malignant populations; ii) the absence of CD138 expression on CLL lymphocytes and the expression of IgD on the surface of the CLL lymphocytes, unlikely lymphoplasmacytic phenotype; iii) detection of IgG instead of IgM paraprotein in the plasma of the patient [15].

The Chromosome 12 is considered as a marker of poor prognosis in B-CLL patients [16], but it was never correlated with myeloma development. Elnenaei and collaborators [17] reported this cytogenetic alteration in 8% of their myeloma patients, but with no informative data about previous hematological disorder, such as B-CLL. We reported the absence of this alteration in plasma cells, suggesting that PCM could have originated independently, from a normal B-cell clone instead of a malignant B-CLL one. We must consider the possibility of PCM has been originated from a chromosome 12-negative clone of B-CLL, or also from a chromosome 12-positive B-CLL clone with subsequent loss of the additional chromosome. They are real possibilities, but not well described in the literature.

Determination of the rearrangements in the immunoglobulin genes is considered the gold standard to infer clonal derivation in B cell neoplasm [18]. Nevertheless, cytogenetic markers may also contribute to understand the clonal relatedness and evolution in concurrent malignant diseases [19]. Deletions, loss of heterozygosis, duplications or translocations occur in several tumor entities and become a molecular legacy in these cells. Kaufmann and colleagues investigated cytogenetic markers in lymphocytes and plasma cells of two patients with concomitant B-CLL and myeloma, and likewise they found no evidence of same clonality for these two diseases [20]. Alternatively, it is clear that treatments for B-CLL may induce transformation of other cells, generating a new tumor entity [21], such as PCM, independently of the B-CLL clones. This case report emphasizes the importance of the close follow up of B-CLL patients for early detection of other malignancies development, such as plasma cell myeloma.

## Consent

Written informed consent was obtained from the patient for publication of this case report. A copy of the written consent is available for review by the Editor-in-Chief of this journal.

## Abbreviations

B-CLL: B cell chronic lymphocytic leukemia; PCM: Plasma cell myeloma; FISH: Fluorescent In Situ hybridization.

## Competing interests

The authors declare that they have no competing interests.

## Authors’ contributions

PWO developed the original idea of the manuscript, carried out the flow cytometry analysis, plasma cell separation, preparation of the figures and wrote the manuscript. BNS participated of the original idea of the manuscript, participated of the flow cytometry analysis. CRP and KRH participated in the flow cytometry analysis and reviewed the manuscript. VED and BD carried out the FISH procedure and analysis and reviewed the manuscript. HN is the responsible for the patient, contributed with clinical data and reviewed the manuscript. CPV participated in the plasma cell separation procedure, figures composition, manuscript writing and reviewed the final version. All authors read and approved the final manuscript.
